# Supplementary material for: Proteotranscriptomic Profiling of the Toxic Mucus of Kulikovia alborostrata (Pilidiophora, Nemertea)
Source: Toxins (Basel). 2024 Dec 26;17(1):5. doi: 10.3390/toxins17010005 (PMC11768458; doi:10.3390/toxins17010005)
Supplement: Supplementary file 1 [file toxins-17-00005-s001.zip › Supplementary File S4.zip!/ORF 006024 LINALB/230688690e3e92c6/~ORF006024.html]

Phyre 2 Results for ORF\_006024\_LINALB


|  |  |
| --- | --- |
|  | Retrieve Phyre Job Id |


|  |  |
| --- | --- |
| Email | vas9i-kz@mail.ru |
| Description | ORF\_006024\_LINALB |
| Date | Fri Apr 7 01:53:58 BST 2023 |
| Unique Job ID | 230688690e3e92c6 |
| Sequence | HEMHDFSEEV ... Download FASTA |
| Job Type | **normal** |
| Job Expiry |  |

|  |  |
| --- | --- |
| Summary |  |

Top model

Image coloured by rainbow N → C terminus

|  |  |
| --- | --- |
| Model dimensions (Å): | **X**:26.234 **Y**:20.742 **Z**:20.544 |

|  |  |
| --- | --- |
|  |  |
 Close JSmol | | |

 Model (left) based on template c6av8A\_ Top template information **PDB header:**toxin **Chain:** A: **PDB Molecule:**u5-theraphotoxin-hs1b 1; **PDBTitle:** exploring cystine dense peptide space to open a unique molecular2 toolbox **PDB Entry:** PDBe RCSB PDBj - Confidence and coverage Confidence: \| **88.4%** \| Coverage: \| **53%** 23 residues ( 53% of your sequence) have been modelled with 88.4% confidence by the single highest scoring template. You may wish to submit your sequence to Phyrealarm. This will automatically scan your sequence every week for new potential templates as they appear in the Phyre2 library. Please note: You must be registered and logged in to use Phyrealarm. 3D viewing Interactive 3D view in JSmol For other options to view your downloaded structure offline see the FAQ |

|  |  |
| --- | --- |
| Sequence analysis |  |

|  |  |
| --- | --- |
|  | Download FASTA version |

|  |  |
| --- | --- |
| Secondary structure and disorder prediction [Show] |  |

|  |  |  |  |
| --- | --- | --- | --- |
|  |  |  |  |

|  |  |  |  |  |  |  |  |  |  |  |  |  |  |  |  |  |  |  |  |  |  |  |  |  |  |  |  |  |  |  |  |  |  |  |  |  |  |  |  |  |  |  |  |  |  |  |  |  |  |  |  |  |  |  |  |  |  |  |  |  |  |  |  |  |  |  |  |  |  |  |  |  |  |  |  |  |  |  |  |  |
| --- | --- | --- | --- | --- | --- | --- | --- | --- | --- | --- | --- | --- | --- | --- | --- | --- | --- | --- | --- | --- | --- | --- | --- | --- | --- | --- | --- | --- | --- | --- | --- | --- | --- | --- | --- | --- | --- | --- | --- | --- | --- | --- | --- | --- | --- | --- | --- | --- | --- | --- | --- | --- | --- | --- | --- | --- | --- | --- | --- | --- | --- | --- | --- | --- | --- | --- | --- | --- | --- | --- | --- | --- | --- | --- | --- | --- | --- | --- | --- | --- |
|  |  | 1 | . | . | . | . | . | . | . | . | 10 | . | . | . | . | . | . | . | . | . | 20 | . | . | . | . | . | . | . | . | . | 30 | . | . | . | . | . | . | . | . | . | 40 | . | . | . |  |  |  |  |  |  |  |  |  |  |  |  |  |  |  |  |  |  |  |  |  |  |  |  |  |  |  |  |  |  |  |  |  |  |  |  |
| Sequence |  | H | E | M | H | D | F | S | E | E | V | S | V | N | K | R | C | S | K | H | G | R | C | G | N | D | Y | D | C | C | P | G | Y | V | C | S | R | K | Y | K | E | C | S | R |  |  |  |  |  |  |  |  |  |  |  |  |  |  |  |  |  |  |  |  |  |  |  |  |  |  |  |  |  |  |  |  |  |  |  |  |
| Secondary structure |  | --- | --- | --- | --- | --- | --- | --- | --- | --- |  |  |  | --- | --- | --- | --- | --- | --- | --- | --- | --- | --- | --- | --- | --- | --- | --- | --- | --- | --- | --- |  |  |  |  | --- | --- | --- | --- | --- | --- | --- | --- |  |  |  |  |  |  |  |  |  |  |  |  |  |  |  |  |  |  |  |  |  |  |  |  |  |  |  |  |  |  |  |  |  |  |  |  |
| SS confidence |  | --- | --- | --- | --- | --- | --- | --- | --- | --- | --- | --- | --- | --- | --- | --- | --- | --- | --- | --- | --- | --- | --- | --- | --- | --- | --- | --- | --- | --- | --- | --- | --- | --- | --- | --- | --- | --- | --- | --- | --- | --- | --- | --- |  |  |  |  |  |  |  |  |  |  |  |  |  |  |  |  |  |  |  |  |  |  |  |  |  |  |  |  |  |  |  |  |  |  |  |  |
| Disorder |  | **?** | **?** | **?** | **?** | **?** | **?** | **?** | **?** | **?** | **?** | **?** | **?** | **?** | **?** | **?** | **?** | **?** | **?** | **?** | **?** | **?** | **?** | **?** | **?** | **?** | **?** | **?** | **?** | **?** | **?** | **?** | **?** | **?** | **?** | **?** | **?** | **?** | **?** | **?** | **?** | **?** | **?** | **?** |  |  |  |  |  |  |  |  |  |  |  |  |  |  |  |  |  |  |  |  |  |  |  |  |  |  |  |  |  |  |  |  |  |  |  |  |
| Disorder confidence |  | --- | --- | --- | --- | --- | --- | --- | --- | --- | --- | --- | --- | --- | --- | --- | --- | --- | --- | --- | --- | --- | --- | --- | --- | --- | --- | --- | --- | --- | --- | --- | --- | --- | --- | --- | --- | --- | --- | --- | --- | --- | --- | --- |  |  |  |  |  |  |  |  |  |  |  |  |  |  |  |  |  |  |  |  |  |  |  |  |  |  |  |  |  |  |  |  |  |  |  |  |
|  | | | | | | | | | | | | | | | | | | | | | | | | | | | | | | | | | | | | | | | | | | | | | | | | | | | | | | | | | | | | | | | | | | | | | | | | | | | | | | | | |

|  |  |  |  |  |  |  |  |  |  |  |  |
| --- | --- | --- | --- | --- | --- | --- | --- | --- | --- | --- | --- |
| Confidence Key | | | | | | | | | | | |
| High(9) |  |  |  |  |  |  |  |  |  |  | Low (0) |

|  |  |
| --- | --- |
| **?** | Disordered ( 44%) |
|  | Alpha helix ( 0%) |
|  |  |

Beta strand ( 16%)

|  |  |
| --- | --- |
| Domain analysis [Show] |  |

|  |  |  |  |
| --- | --- | --- | --- |
|  |  |  |  |

|  |
| --- |
| (Rank \| Aligned region) |
| (1 \| (--- \| c6av8A\_ \| ---); PDB 6av8 chain A **Region:** 21 - 43 **Aligned:** 23 **Modelled:** 23 **Confidence:** 88.4% **Identity:** 48% **PDB header:**toxin **Chain:** A: **PDB Molecule:**u5-theraphotoxin-hs1b 1; **PDBTitle:** exploring cystine dense peptide space to open a unique molecular2 toolbox **PDB Entry:** PDBe RCSB PDBj Phyre2; 2 \| (--- \| c7rz3A\_ \| ---); PDB 7rz3 chain A **Region:** 25 - 43 **Aligned:** 19 **Modelled:** 19 **Confidence:** 38.5% **Identity:** 58% **PDB header:**toxin **Chain:** A: **PDB Molecule:**xt3a; **PDBTitle:** the solution structure of remipede double-ick toxin phi-xibalbin3-xt3a **PDB Entry:** PDBe RCSB PDBj Phyre2; 3 \| (--- \| c5t4rA\_ \| ---); PDB 5t4r chain A **Region:** 20 - 41 **Aligned:** 22 **Modelled:** 22 **Confidence:** 51.3% **Identity:** 50% **PDB header:**toxin **Chain:** A: **PDB Molecule:**mu-theraphotoxin-pn3a; **PDBTitle:** nmr solution structure of the nav1.7 selective spider venom-derived2 peptide pn3a **PDB Entry:** PDBe RCSB PDBj Phyre2; 4 \| (--- \| c5we3A\_ \| ---); PDB 5we3 chain A **Region:** 24 - 41 **Aligned:** 18 **Modelled:** 18 **Confidence:** 42.1% **Identity:** 61% **PDB header:**toxin **Chain:** A: **PDB Molecule:**beta-theraphotoxin-ps1a; **PDBTitle:** solution nmr structure of paurtx-3 **PDB Entry:** PDBe RCSB PDBj Phyre2; 5 \| (--- \| d1qk7a\_ \| ---); PDB 1qk7 chain A **Region:** 21 - 41 **Aligned:** 21 **Modelled:** 21 **Confidence:** 94.6% **Identity:** 52% **Fold:** Knottins (small inhibitors, toxins, lectins) **Superfamily:** omega toxin-like **Family:** Spider toxins Phyre2; 6 \| (--- \| c6f61A\_ \| ---); PDB 6f61 chain A **Region:** 16 - 41 **Aligned:** 26 **Modelled:** 26 **Confidence:** 50.5% **Identity:** 42% **PDB header:**antimicrobial protein **Chain:** A: **PDB Molecule:**purotoxin-6; **PDBTitle:** nmr structure of purotoxin-6 **PDB Entry:** PDBe RCSB PDBj Phyre2; 7 \| (--- \| c7skcA\_ \| ---); PDB 7skc chain A **Region:** 24 - 41 **Aligned:** 18 **Modelled:** 18 **Confidence:** 84.5% **Identity:** 56% **PDB header:**toxin **Chain:** A: **PDB Molecule:**ssp1a toxin; **PDBTitle:** solution structure of spider toxin ssp1a **PDB Entry:** PDBe RCSB PDBj Phyre2; 8 \| (--- \| c1s6xA\_ \| ---); PDB 1s6x chain A **Region:** 21 - 41 **Aligned:** 21 **Modelled:** 21 **Confidence:** 74.1% **Identity:** 48% **PDB header:**toxin **Chain:** A: **PDB Molecule:**kvap channel; **PDBTitle:** solution structure of vstx **PDB Entry:** PDBe RCSB PDBj Phyre2; 9 \| (--- \| c1qfdA\_ \| ---); PDB 1qfd chain A **Region:** 18 - 38 **Aligned:** 21 **Modelled:** 21 **Confidence:** 16.4% **Identity:** 48% **PDB header:**inhibitor **Chain:** A: **PDB Molecule:**protein (alpha-amylase inhibitor); **PDBTitle:** nmr solution structure of alpha-amylase inhibitor (aai) **PDB Entry:** PDBe RCSB PDBj Phyre2; 10 \| (--- \| d1clvi\_ \| ---); PDB 1clv chain I **Region:** 18 - 38 **Aligned:** 21 **Modelled:** 20 **Confidence:** 13.6% **Identity:** 48% **Fold:** Knottins (small inhibitors, toxins, lectins) **Superfamily:** Plant inhibitors of proteinases and amylases **Family:** Plant inhibitors of proteinases and amylases Phyre2; 11 \| (--- \| c8feyA\_ \| ---); PDB 8fey chain A **Region:** 20 - 38 **Aligned:** 19 **Modelled:** 19 **Confidence:** 77.7% **Identity:** 53% **PDB header:**toxin **Chain:** A: **PDB Molecule:**pmu1a toxin; **PDBTitle:** solution structure of pmu1a **PDB Entry:** PDBe RCSB PDBj Phyre2; 12 \| (--- \| d1en2a2 \| ---); PDB 1en2 chain A domain 2 **Region:** 16 - 34 **Aligned:** 19 **Modelled:** 19 **Confidence:** 30.9% **Identity:** 53% **Fold:** Knottins (small inhibitors, toxins, lectins) **Superfamily:** Plant lectins/antimicrobial peptides **Family:** Hevein-like agglutinin (lectin) domain Phyre2; 13 \| (--- \| d1ehda2 \| ---); PDB 1ehd chain A domain 2 **Region:** 16 - 34 **Aligned:** 19 **Modelled:** 19 **Confidence:** 29.3% **Identity:** 53% **Fold:** Knottins (small inhibitors, toxins, lectins) **Superfamily:** Plant lectins/antimicrobial peptides **Family:** Hevein-like agglutinin (lectin) domain Phyre2; 14 \| (--- \| c7namB\_ \| ---); PDB 7nam chain B **Region:** 18 - 34 **Aligned:** 17 **Modelled:** 17 **Confidence:** 73.8% **Identity:** 59% **PDB header:**signaling protein **Chain:** B: **PDB Molecule:**trypsin inhibitor 2; **PDBTitle:** lrp6\_e1 in complex with lr-eet-3.5 **PDB Entry:** PDBe RCSB PDBj Phyre2; 15 \| (--- \| c6f8eA\_ \| ---); PDB 6f8e chain A **Region:** 4 - 22 **Aligned:** 17 **Modelled:** 19 **Confidence:** 12.7% **Identity:** 59% **PDB header:**lipid binding protein **Chain:** A: **PDB Molecule:**pleckstrin homology domain; **PDBTitle:** ph domain from tgaph **PDB Entry:** PDBe RCSB PDBj Phyre2; 16 \| (--- \| c2n86A\_ \| ---); PDB 2n86 chain A **Region:** 10 - 34 **Aligned:** 25 **Modelled:** 25 **Confidence:** 15.3% **Identity:** 40% **PDB header:**toxin **Chain:** A: **PDB Molecule:**spiderine-1a; **PDBTitle:** nmr structure of ottx1a - ick **PDB Entry:** PDBe RCSB PDBj Phyre2; 17 \| (--- \| c1zwuA\_ \| ---); PDB 1zwu chain A **Region:** 20 - 43 **Aligned:** 23 **Modelled:** 24 **Confidence:** 9.4% **Identity:** 43% **PDB header:**antimicrobial protein **Chain:** A: **PDB Molecule:**amaranthus caudatus antimicrobial peptide 2 (acmp2); **PDBTitle:** 30 nmr structures of acamp2-like peptide with non natural beta-(2-2 naphthyl)-alanine residue. **PDB Entry:** PDBe RCSB PDBj Phyre2; 18 \| (--- \| c2m9lA\_ \| ---); PDB 2m9l chain A **Region:** 20 - 41 **Aligned:** 22 **Modelled:** 22 **Confidence:** 57.4% **Identity:** 41% **PDB header:**toxin **Chain:** A: **PDB Molecule:**beta-theraphotoxin-tp1a; **PDBTitle:** solution structure of protoxin-1 **PDB Entry:** PDBe RCSB PDBj Phyre2; 19 \| (--- \| c2a2vA\_ \| ---); PDB 2a2v chain A **Region:** 20 - 41 **Aligned:** 22 **Modelled:** 22 **Confidence:** 23.0% **Identity:** 41% **PDB header:**toxin **Chain:** A: **PDB Molecule:**jingzhaotoxin-xi; **PDBTitle:** the solution structure of jingzhaotoxin-xi **PDB Entry:** PDBe RCSB PDBj Phyre2; 20 \| (--- \| c2jtbA\_ \| ---); PDB 2jtb chain A **Region:** 27 - 41 **Aligned:** 15 **Modelled:** 15 **Confidence:** 77.1% **Identity:** 60% **PDB header:**toxin **Chain:** A: **PDB Molecule:**hainantoxin-3; **PDBTitle:** three dimensional solution structure of hainantoxin-iii by 2d 1h-nmr **PDB Entry:** PDBe RCSB PDBj Phyre2; 21 \| (--- \| --- \| ---); 22 \| (--- \| --- \| ---); 23 \| (--- \| --- \| ---); 24 \| (--- \| --- \| ---); 25 \| (--- \| --- \| ---); 26 \| (--- \| --- \| ---); 27 \| (--- \| --- \| ---); 28 \| (--- \| --- \| ---); 29 \| (--- \| --- \| ---); 30 \| (--- \| --- \| ---); 31 \| (--- \| --- \| ---); 32 \| (--- \| --- \| ---); 33 \| (--- \| --- \| ---); 34 \| (--- \| --- \| ---); 35 \| (--- \| --- \| ---); 36 \| (--- \| --- \| ---); 37 \| (--- \| --- \| ---); 38 \| (--- \| --- \| ---); 39 \| (--- \| --- \| ---); 40 \| (--- \| --- \| ---); 41 \| (--- \| --- \| ---); 42 \| (--- \| --- \| ---); 43 \| (--- \| --- \| ---); 44 \| (--- \| --- \| ---); 45 \| (--- \| --- \| ---); 46 \| (--- \| --- \| ---); 47 \| (--- \| --- \| ---); 48 \| (--- \| --- \| ---); 49 \| (--- \| --- \| ---); 50 \| (--- \| --- \| ---); 51 \| (--- \| --- \| ---); 52 \| (--- \| --- \| ---); 53 \| (--- \| --- \| ---); 54 \| (--- \| --- \| ---); 55 \| (--- \| --- \| ---); 56 \| (--- \| --- \| ---); 57 \| (--- \| --- \| ---); 58 \| (--- \| --- \| ---); 59 \| (--- \| --- \| ---); 60 \| (--- \| --- \| ---); 61 \| (--- \| --- \| ---); 62 \| (--- \| --- \| ---); 63 \| (--- \| --- \| ---); 64 \| (--- \| --- \| ---); 65 \| (--- \| --- \| ---); 66 \| (--- \| --- \| ---); 67 \| (--- \| --- \| ---); 68 \| (--- \| --- \| ---); 69 \| (--- \| --- \| ---); 70 \| (--- \| --- \| ---); 71 \| (--- \| --- \| ---); 72 \| (--- \| --- \| ---); 73 \| (--- \| --- \| ---); 74 \| (--- \| --- \| ---); 75 \| (--- \| --- \| ---); 76 \| (--- \| --- \| ---); 77 \| (--- \| --- \| ---); 78 \| (--- \| --- \| ---); 79 \| (--- \| --- \| ---); 80 \| (--- \| --- \| ---); 81 \| (--- \| --- \| ---); 82 \| (--- \| --- \| ---); 83 \| (--- \| --- \| ---); 84 \| (--- \| --- \| ---); 85 \| (--- \| --- \| ---); 86 \| (--- \| --- \| ---); 87 \| (--- \| --- \| ---); 88 \| (--- \| --- \| ---); 89 \| (--- \| --- \| ---); 90 \| (--- \| --- \| ---); 91 \| (--- \| --- \| ---); 92 \| (--- \| --- \| ---); 93 \| (--- \| --- \| ---); 94 \| (--- \| --- \| ---); 95 \| (--- \| --- \| ---); 96 \| (--- \| --- \| ---); 97 \| (--- \| --- \| ---); 98 \| (--- \| --- \| ---); 99 \| (--- \| --- \| ---)) |

|  |  |
| --- | --- |
| Detailed template information [Hide] |  |

|  |  |  |  |
| --- | --- | --- | --- |
|  |  |  |  |

| # | Template | Alignment Coverage | 3D Model | Confidence | % i.d. | Template Information |
| --- | --- | --- | --- | --- | --- | --- |

|  |  |  |  |  |  |  |
| --- | --- | --- | --- | --- | --- | --- |
| 1 | c6av8A\_ | --- \| --- \| --- |  | 88.4 | 48 |


 (**PDB header:**toxin **Chain:** A: **PDB Molecule:**u5-theraphotoxin-hs1b 1; **PDBTitle:** exploring cystine dense peptide space to open a unique molecular2 toolbox **PDB Entry:** PDBe RCSB PDBj) |
| 2 | c7rz3A\_ | --- \| --- \| --- |  | 38.5 | 58 |


 (**PDB header:**toxin **Chain:** A: **PDB Molecule:**xt3a; **PDBTitle:** the solution structure of remipede double-ick toxin phi-xibalbin3-xt3a **PDB Entry:** PDBe RCSB PDBj) |
| 3 | c5t4rA\_ | --- \| --- \| --- |  | 51.3 | 50 |


 (**PDB header:**toxin **Chain:** A: **PDB Molecule:**mu-theraphotoxin-pn3a; **PDBTitle:** nmr solution structure of the nav1.7 selective spider venom-derived2 peptide pn3a **PDB Entry:** PDBe RCSB PDBj) |
| 4 | c5we3A\_ | --- \| --- \| --- |  | 42.1 | 61 |


 (**PDB header:**toxin **Chain:** A: **PDB Molecule:**beta-theraphotoxin-ps1a; **PDBTitle:** solution nmr structure of paurtx-3 **PDB Entry:** PDBe RCSB PDBj) |
| 5 | d1qk7a\_ | --- \| --- \| --- |  | 94.6 | 52 | **Fold:** Knottins (small inhibitors, toxins, lectins) **Superfamily:** omega toxin-like **Family:** Spider toxins **PDB entry:** PDBe RCSB PDBj |
| 6 | c6f61A\_ | --- \| --- \| --- |  | 50.5 | 42 |


 (**PDB header:**antimicrobial protein **Chain:** A: **PDB Molecule:**purotoxin-6; **PDBTitle:** nmr structure of purotoxin-6 **PDB Entry:** PDBe RCSB PDBj) |
| 7 | c7skcA\_ | --- \| --- \| --- |  | 84.5 | 56 |


 (**PDB header:**toxin **Chain:** A: **PDB Molecule:**ssp1a toxin; **PDBTitle:** solution structure of spider toxin ssp1a **PDB Entry:** PDBe RCSB PDBj) |
| 8 | c1s6xA\_ | --- \| --- \| --- |  | 74.1 | 48 |


 (**PDB header:**toxin **Chain:** A: **PDB Molecule:**kvap channel; **PDBTitle:** solution structure of vstx **PDB Entry:** PDBe RCSB PDBj) |
| 9 | c1qfdA\_ | --- \| --- \| --- |  | 16.4 | 48 |


 (**PDB header:**inhibitor **Chain:** A: **PDB Molecule:**protein (alpha-amylase inhibitor); **PDBTitle:** nmr solution structure of alpha-amylase inhibitor (aai) **PDB Entry:** PDBe RCSB PDBj) |
| 10 | d1clvi\_ | --- \| --- \| --- |  | 13.6 | 48 | **Fold:** Knottins (small inhibitors, toxins, lectins) **Superfamily:** Plant inhibitors of proteinases and amylases **Family:** Plant inhibitors of proteinases and amylases **PDB entry:** PDBe RCSB PDBj |
| 11 | c8feyA\_ | --- \| --- \| --- |  | 77.7 | 53 |


 (**PDB header:**toxin **Chain:** A: **PDB Molecule:**pmu1a toxin; **PDBTitle:** solution structure of pmu1a **PDB Entry:** PDBe RCSB PDBj) |
| 12 | d1en2a2 | --- \| --- \| --- |  | 30.9 | 53 | **Fold:** Knottins (small inhibitors, toxins, lectins) **Superfamily:** Plant lectins/antimicrobial peptides **Family:** Hevein-like agglutinin (lectin) domain **PDB entry:** PDBe RCSB PDBj |
| 13 | d1ehda2 | --- \| --- \| --- |  | 29.3 | 53 | **Fold:** Knottins (small inhibitors, toxins, lectins) **Superfamily:** Plant lectins/antimicrobial peptides **Family:** Hevein-like agglutinin (lectin) domain **PDB entry:** PDBe RCSB PDBj |
| 14 | c7namB\_ | --- \| --- \| --- |  | 73.8 | 59 |


 (**PDB header:**signaling protein **Chain:** B: **PDB Molecule:**trypsin inhibitor 2; **PDBTitle:** lrp6\_e1 in complex with lr-eet-3.5 **PDB Entry:** PDBe RCSB PDBj) |
| 15 | c6f8eA\_ | --- \| --- \| --- |  | 12.7 | 59 |


 (**PDB header:**lipid binding protein **Chain:** A: **PDB Molecule:**pleckstrin homology domain; **PDBTitle:** ph domain from tgaph **PDB Entry:** PDBe RCSB PDBj) |
| 16 | c2n86A\_ | --- \| --- \| --- |  | 15.3 | 40 |


 (**PDB header:**toxin **Chain:** A: **PDB Molecule:**spiderine-1a; **PDBTitle:** nmr structure of ottx1a - ick **PDB Entry:** PDBe RCSB PDBj) |
| 17 | c1zwuA\_ | --- \| --- \| --- |  | 9.4 | 43 |


 (**PDB header:**antimicrobial protein **Chain:** A: **PDB Molecule:**amaranthus caudatus antimicrobial peptide 2 (acmp2); **PDBTitle:** 30 nmr structures of acamp2-like peptide with non natural beta-(2-2 naphthyl)-alanine residue. **PDB Entry:** PDBe RCSB PDBj) |
| 18 | c2m9lA\_ | --- \| --- \| --- |  | 57.4 | 41 |


 (**PDB header:**toxin **Chain:** A: **PDB Molecule:**beta-theraphotoxin-tp1a; **PDBTitle:** solution structure of protoxin-1 **PDB Entry:** PDBe RCSB PDBj) |
| 19 | c2a2vA\_ | --- \| --- \| --- |  | 23.0 | 41 |


 (**PDB header:**toxin **Chain:** A: **PDB Molecule:**jingzhaotoxin-xi; **PDBTitle:** the solution structure of jingzhaotoxin-xi **PDB Entry:** PDBe RCSB PDBj) |
| 20 | c2jtbA\_ | --- \| --- \| --- |  | 77.1 | 60 |


 (**PDB header:**toxin **Chain:** A: **PDB Molecule:**hainantoxin-3; **PDBTitle:** three dimensional solution structure of hainantoxin-iii by 2d 1h-nmr **PDB Entry:** PDBe RCSB PDBj) |
| 21 | c1qk6A\_ | --- \| --- \| --- | not modelled | 58.7 | 50 |


 (**PDB header:**toxin **Chain:** A: **PDB Molecule:**huwentoxin-i; **PDBTitle:** solution structure of huwentoxin-i by nmr **PDB Entry:** PDBe RCSB PDBj) |
| 22 | c7jpmA\_ | --- \| --- \| --- | not modelled | 21.7 | 50 |


 (**PDB header:**toxin **Chain:** A: **PDB Molecule:**omega-theraphotoxin-pm1b; **PDBTitle:** the solution structure of omega-theraphotoxin-pm1b isolated from king2 baboon spider **PDB Entry:** PDBe RCSB PDBj) |
| 23 | c6mk5A\_ | --- \| --- \| --- | not modelled | 21.3 | 50 |


 (**PDB header:**toxin **Chain:** A: **PDB Molecule:**toxin gtx1-15; **PDBTitle:** solution nmr structure of spider toxin analogue [f5a,m6f,t26l,2 k28r]gptx-1 **PDB Entry:** PDBe RCSB PDBj) |
| 24 | d1tyka\_ | --- \| --- \| --- | not modelled | 9.6 | 50 | **Fold:** Knottins (small inhibitors, toxins, lectins) **Superfamily:** omega toxin-like **Family:** Spider toxins **PDB entry:** PDBe RCSB PDBj |
| 25 | c2wh9A\_ | --- \| --- \| --- | not modelled | 90.0 | 64 |


 (**PDB header:**membrane protein inhibitor **Chain:** A: **PDB Molecule:**guangxitoxin-1egxtx-1e; **PDBTitle:** solution structure of gxtx-1e **PDB Entry:** PDBe RCSB PDBj) |
| 26 | d1eita\_ | --- \| --- \| --- | not modelled | 34.6 | 56 | **Fold:** Knottins (small inhibitors, toxins, lectins) **Superfamily:** omega toxin-like **Family:** Spider toxins **PDB entry:** PDBe RCSB PDBj |
| 27 | c1eitA\_ | --- \| --- \| --- | not modelled | 34.6 | 56 |


 (**PDB header:**neurotoxin **Chain:** A: **PDB Molecule:**mu-agatoxin-i; **PDBTitle:** nmr study of mu-agatoxin **PDB Entry:** PDBe RCSB PDBj) |
| 28 | c2aapA\_ | --- \| --- \| --- | not modelled | 40.6 | 47 |


 (**PDB header:**toxin **Chain:** A: **PDB Molecule:**jingzhaotoxin-vii; **PDBTitle:** solution structure of jingzhaotoxin-vii **PDB Entry:** PDBe RCSB PDBj) |
| 29 | c1emxA\_ | --- \| --- \| --- | not modelled | 67.9 | 62 |


 (**PDB header:**toxin **Chain:** A: **PDB Molecule:**heteropodatoxin 2; **PDBTitle:** solution structure of hptx2, a toxin from heteropoda venatoria spider2 venom that blocks kv4.2 potassium channel **PDB Entry:** PDBe RCSB PDBj) |
| 30 | c6ceiA\_ | --- \| --- \| --- | not modelled | 75.0 | 50 |


 (**PDB header:**toxin **Chain:** A: **PDB Molecule:**gxia; **PDBTitle:** solution nmr structure of conotoxin gxia from conus geographus **PDB Entry:** PDBe RCSB PDBj) |
| 31 | c2n3pA\_ | --- \| --- \| --- | not modelled | 13.5 | 32 |


 (**PDB header:**toxin **Chain:** A: **PDB Molecule:**asteropsin\_g; **PDBTitle:** solution nmr structure of asteropsin g from marine sponge asteropus **PDB Entry:** PDBe RCSB PDBj) |
| 32 | c5yjhA\_ | --- \| --- \| --- | not modelled | 25.5 | 47 |


 (**PDB header:**cell adhesion **Chain:** A: **PDB Molecule:**periostin; **PDBTitle:** structural insights into periostin functions **PDB Entry:** PDBe RCSB PDBj) |
| 33 | c6f24A\_ | --- \| --- \| --- | not modelled | 11.7 | 47 |


 (**PDB header:**lipid binding protein **Chain:** A: **PDB Molecule:**c-terminal ph domain from pfaph; **PDBTitle:** ph domain from pfaph **PDB Entry:** PDBe RCSB PDBj) |
| 34 | c5nv6A\_ | --- \| --- \| --- | not modelled | 10.0 | 47 |


 (**PDB header:**structural protein **Chain:** A: **PDB Molecule:**transforming growth factor-beta-induced protein ig-h3; **PDBTitle:** structure of human transforming growth factor beta-induced protein2 (tgfbip). **PDB Entry:** PDBe RCSB PDBj) |
| 35 | c1kozA\_ | --- \| --- \| --- | not modelled | 44.7 | 42 |


 (**PDB header:**toxin **Chain:** A: **PDB Molecule:**voltage-dependent channel inhibitor; **PDBTitle:** solution structure of omega-grammotoxin sia **PDB Entry:** PDBe RCSB PDBj) |
| 36 | c1mcvI\_ | --- \| --- \| --- | not modelled | 22.2 | 57 |


 (**PDB header:**hydrolase **Chain:** I: **PDB Molecule:**hei-toe i; **PDBTitle:** crystal structure analysis of a hybrid squash inhibitor in2 complex with porcine pancreatic elastase **PDB Entry:** PDBe RCSB PDBj) |
| 37 | d1mcvi\_ | --- \| --- \| --- | not modelled | 22.2 | 57 | **Fold:** Knottins (small inhibitors, toxins, lectins) **Superfamily:** Plant inhibitors of proteinases and amylases **Family:** Plant inhibitors of proteinases and amylases **PDB entry:** PDBe RCSB PDBj |
| 38 | d2it7a1 | --- \| --- \| --- | not modelled | 21.9 | 57 | **Fold:** Knottins (small inhibitors, toxins, lectins) **Superfamily:** Plant inhibitors of proteinases and amylases **Family:** Plant inhibitors of proteinases and amylases **PDB entry:** PDBe RCSB PDBj |
| 39 | c2it7A\_ | --- \| --- \| --- | not modelled | 21.9 | 57 |


 (**PDB header:**plant protein **Chain:** A: **PDB Molecule:**trypsin inhibitor 2; **PDBTitle:** solution structure of the squash trypsin inhibitor eeti-ii **PDB Entry:** PDBe RCSB PDBj) |
| 40 | c2letA\_ | --- \| --- \| --- | not modelled | 21.7 | 57 |


 (**PDB header:**proteinase inhibitor(trypsin) **Chain:** A: **PDB Molecule:**trypsin inhibitor ii; **PDBTitle:** an 1h nmr determination of the three dimensional structures of mirror2 image forms of a leu-5 variant of the trypsin inhibitor ecballium3 elaterium (eeti-ii) **PDB Entry:** PDBe RCSB PDBj) |
| 41 | d1h9hi\_ | --- \| --- \| --- | not modelled | 20.3 | 57 | **Fold:** Knottins (small inhibitors, toxins, lectins) **Superfamily:** Plant inhibitors of proteinases and amylases **Family:** Plant inhibitors of proteinases and amylases **PDB entry:** PDBe RCSB PDBj |
| 42 | c6a48A\_ | --- \| --- \| --- | not modelled | 13.9 | 42 |


 (**PDB header:**signaling protein **Chain:** A: **PDB Molecule:**reelin; **PDBTitle:** crystal structure of reelin n-terminal region **PDB Entry:** PDBe RCSB PDBj) |
| 43 | c6gftA\_ | --- \| --- \| --- | not modelled | 26.3 | 53 |


 (**PDB header:**toxin **Chain:** A: **PDB Molecule:**cyriotoxin-1a; **PDBTitle:** antinociceptive evaluation of cyriotoxin-1a, the first toxin purified2 from cyriopagopus schioedtei spider venom **PDB Entry:** PDBe RCSB PDBj) |
| 44 | d1h9ii\_ | --- \| --- \| --- | not modelled | 21.6 | 53 | **Fold:** Knottins (small inhibitors, toxins, lectins) **Superfamily:** Plant inhibitors of proteinases and amylases **Family:** Plant inhibitors of proteinases and amylases **PDB entry:** PDBe RCSB PDBj |
| 45 | c5i1xA\_ | --- \| --- \| --- | not modelled | 59.8 | 44 |


 (**PDB header:**toxin **Chain:** A: **PDB Molecule:**mu-trtx-pre1a toxin; **PDBTitle:** resonance assignments and nmr structure determination of tarantula2 toxin, f8a mutant of beta-trtx-pre1a **PDB Entry:** PDBe RCSB PDBj) |
| 46 | c2mt7A\_ | --- \| --- \| --- | not modelled | 56.3 | 44 |


 (**PDB header:**toxin **Chain:** A: **PDB Molecule:**hs1a; **PDBTitle:** solution structure of spider-venom peptide hs1a **PDB Entry:** PDBe RCSB PDBj) |
| 47 | c5o57A\_ | --- \| --- \| --- | not modelled | 21.1 | 44 |


 (**PDB header:**signaling protein **Chain:** A: **PDB Molecule:**dickkopf-related protein 4; **PDBTitle:** solution structure of the n-terminal region of dkk4 **PDB Entry:** PDBe RCSB PDBj) |
| 48 | d1d1ha\_ | --- \| --- \| --- | not modelled | 16.2 | 36 | **Fold:** Knottins (small inhibitors, toxins, lectins) **Superfamily:** omega toxin-like **Family:** Spider toxins **PDB entry:** PDBe RCSB PDBj |
| 49 | d1la4a\_ | --- \| --- \| --- | not modelled | 16.1 | 36 | **Fold:** Knottins (small inhibitors, toxins, lectins) **Superfamily:** omega toxin-like **Family:** Spider toxins **PDB entry:** PDBe RCSB PDBj |
| 50 | c2kusA\_ | --- \| --- \| --- | not modelled | 12.2 | 36 |


 (**PDB header:**antimicrobial protein **Chain:** A: **PDB Molecule:**sm-amp-1.1a; **PDBTitle:** spatial structure of antimicrobial peptide sm-amp-1.1a **PDB Entry:** PDBe RCSB PDBj) |
| 51 | c2mvaA\_ | --- \| --- \| --- | not modelled | 11.2 | 44 |


 (**PDB header:**toxin **Chain:** A: **PDB Molecule:**rhtx toxin; **PDBTitle:** solution structure of the toxin, rhtx **PDB Entry:** PDBe RCSB PDBj) |
| 52 | d1nixa\_ | --- \| --- \| --- | not modelled | 20.7 | 47 | **Fold:** Knottins (small inhibitors, toxins, lectins) **Superfamily:** omega toxin-like **Family:** Spider toxins **PDB entry:** PDBe RCSB PDBj |
| 53 | c6nk9A\_ | --- \| --- \| --- | not modelled | 15.6 | 47 |


 (**PDB header:**toxin **Chain:** A: **PDB Molecule:**aca toxin 1; **PDBTitle:** solution structure of acatx1, a potassium channel inhibitor from the2 sea anemone antopleura cascaia **PDB Entry:** PDBe RCSB PDBj) |
| 54 | c2jyeA\_ | --- \| --- \| --- | not modelled | 20.5 | 32 |


 (**PDB header:**cytokine **Chain:** A: **PDB Molecule:**granulin a; **PDBTitle:** human granulin a **PDB Entry:** PDBe RCSB PDBj) |
| 55 | c6v6tA\_ | --- \| --- \| --- | not modelled | 14.1 | 32 |


 (**PDB header:**toxin **Chain:** A: **PDB Molecule:**delta-theraphotoxin-hm1b; **PDBTitle:** solution structure of delta-theraphotoxin-hm1b from heteroscodra2 maculata **PDB Entry:** PDBe RCSB PDBj) |
| 56 | c6btvA\_ | --- \| --- \| --- | not modelled | 69.8 | 39 |


 (**PDB header:**toxin **Chain:** A: **PDB Molecule:**beta-theraphotoxin-cm1b; **PDBTitle:** solution nmr structures for ccotx-ii **PDB Entry:** PDBe RCSB PDBj) |
| 57 | c5epmC\_ | --- \| --- \| --- | not modelled | 41.7 | 39 |


 (**PDB header:**toxin/immune system **Chain:** C: **PDB Molecule:**beta-theraphotoxin-cm1a; **PDBTitle:** ceratotoxin variant in complex with specific antibody fab fragment **PDB Entry:** PDBe RCSB PDBj) |
| 58 | c2i1tA\_ | --- \| --- \| --- | not modelled | 37.9 | 50 |


 (**PDB header:**toxin **Chain:** A: **PDB Molecule:**jingzhaotoxin-3; **PDBTitle:** solution structure of jingzhaotoxin-iii, a novel toxin inhibiting both2 nav and kv channels **PDB Entry:** PDBe RCSB PDBj) |
| 59 | c5ki9A\_ | --- \| --- \| --- | not modelled | 31.1 | 70 |


 (**PDB header:**antimicrobial protein **Chain:** A: **PDB Molecule:**beta-defensin 104; **PDBTitle:** crystal structure of human beta-defensin 4 (hbd4) **PDB Entry:** PDBe RCSB PDBj) |
| 60 | c6lnrA\_ | --- \| --- \| --- | not modelled | 15.4 | 50 |


 (**PDB header:**hydrolase **Chain:** A: **PDB Molecule:**class-1 chitinase; **PDBTitle:** structure of intact chitinase with hevein domain from the plant2 simarouba glauca, known for its traditional anti-inflammatory3 efficacy **PDB Entry:** PDBe RCSB PDBj) |
| 61 | c2ys0A\_ | --- \| --- \| --- | not modelled | 10.3 | 50 |


 **PDB header:**hydrolase **Chain:** A: **PDB Molecule:**ectonucleotide pyrophosphatase/phosphodiesterase family **PDBTitle:** solution structure of the somatomedin b domain of human ectonucleotide2 pyrophosphatase/phosphodiesterase family member **PDB Entry:** PDBe RCSB PDBj |
| 62 | c6ji7A\_ | --- \| --- \| --- | not modelled | 55.8 | 58 |


 (**PDB header:**plant protein **Chain:** A: **PDB Molecule:**coffeetide; **PDBTitle:** coffeetides: iron-binding cysteine rich peptides from coffee waste **PDB Entry:** PDBe RCSB PDBj) |
| 63 | c1omgA\_ | --- \| --- \| --- | not modelled | 15.3 | 58 |


 (**PDB header:**presynaptic neurotoxin **Chain:** A: **PDB Molecule:**omega-conotoxin mviia; **PDBTitle:** nmr study of omega-conotoxin mviia **PDB Entry:** PDBe RCSB PDBj) |
| 64 | c1dw4A\_ | --- \| --- \| --- | not modelled | 15.3 | 58 |


 (**PDB header:**toxin **Chain:** A: **PDB Molecule:**omega-conotoxin mviia; **PDBTitle:** nmr structure of omega-conotoxin mviia: constraints on disulphide2 bridges **PDB Entry:** PDBe RCSB PDBj) |
| 65 | c7mixB\_ | --- \| --- \| --- | not modelled | 15.3 | 58 |


 (**PDB header:**transport protein/toxin **Chain:** B: **PDB Molecule:**omega-conotoxin mviia; **PDBTitle:** human n-type voltage-gated calcium channel cav2.2 in the presence of2 ziconotide at 3.0 angstrom resolution **PDB Entry:** PDBe RCSB PDBj) |
| 66 | c1tt3A\_ | --- \| --- \| --- | not modelled | 15.2 | 58 |


 (**PDB header:**toxin **Chain:** A: **PDB Molecule:**omega-conotoxin mviia; **PDBTitle:** nmr soulution structure of omega-conotoxin [k10]mviia **PDB Entry:** PDBe RCSB PDBj) |
| 67 | c1mviA\_ | --- \| --- \| --- | not modelled | 15.1 | 58 |


 (**PDB header:**neurotoxin **Chain:** A: **PDB Molecule:**mviia; **PDBTitle:** n-type calcium channel blocker, omega-conotoxin mviia, nmr, 152 structures **PDB Entry:** PDBe RCSB PDBj) |
| 68 | c1dw5A\_ | --- \| --- \| --- | not modelled | 15.1 | 58 |


 (**PDB header:**toxin **Chain:** A: **PDB Molecule:**omega-conotoxin mviia; **PDBTitle:** nmr structure of omega-conotoxin mviia: no constraints on disulphide2 bridges **PDB Entry:** PDBe RCSB PDBj) |
| 69 | c7vfuE\_ | --- \| --- \| --- | not modelled | 15.0 | 58 |


 (**PDB header:**membrane protein **Chain:** E: **PDB Molecule:**omega-conotoxin mviia; **PDBTitle:** human n-type voltage gated calcium channel cav2.2-alpha2/delta1-beta12 complex, bound to ziconotide **PDB Entry:** PDBe RCSB PDBj) |
| 70 | c1ttkA\_ | --- \| --- \| --- | not modelled | 14.9 | 58 |


 (**PDB header:**toxin **Chain:** A: **PDB Molecule:**omega-conotoxin mviia; **PDBTitle:** nmr solution structure of omega-conotoxin mviia, a n-type calcium2 channel blocker **PDB Entry:** PDBe RCSB PDBj) |
| 71 | c1feoA\_ | --- \| --- \| --- | not modelled | 14.1 | 58 |


 (**PDB header:**toxin **Chain:** A: **PDB Molecule:**omega-conotoxin mviia-gly; **PDBTitle:** solution structure of omega-conotoxin mviia with c-terminal gly **PDB Entry:** PDBe RCSB PDBj) |
| 72 | c3s8vX\_ | --- \| --- \| --- | not modelled | 11.8 | 38 |


 (**PDB header:**signaling protein **Chain:** X: **PDB Molecule:**dickkopf-related protein 1; **PDBTitle:** crystal structure of lrp6-dkk1 complex **PDB Entry:** PDBe RCSB PDBj) |
| 73 | d1hp3a\_ | --- \| --- \| --- | not modelled | 10.6 | 55 | **Fold:** Knottins (small inhibitors, toxins, lectins) **Superfamily:** omega toxin-like **Family:** Spider toxins **PDB entry:** PDBe RCSB PDBj |
| 74 | c1hp3A\_ | --- \| --- \| --- | not modelled | 10.6 | 55 |


 (**PDB header:**toxin **Chain:** A: **PDB Molecule:**omega-atracotoxin-hv2a; **PDBTitle:** c-terminal truncation of omega-atracotoxin-hv2a (ct-hv2a) **PDB Entry:** PDBe RCSB PDBj) |
| 75 | c2dcwA\_ | --- \| --- \| --- | not modelled | 9.9 | 55 |


 (**PDB header:**antimicrobial protein **Chain:** A: **PDB Molecule:**tachystatin-b2; **PDBTitle:** the solution structure of horseshoe crab antimicrobial peptide2 tachystatin b with the inhibitory cystine-knot motif **PDB Entry:** PDBe RCSB PDBj) |
| 76 | c2dcvA\_ | --- \| --- \| --- | not modelled | 9.8 | 55 |


 (**PDB header:**antimicrobial protein **Chain:** A: **PDB Molecule:**tachystatin-b1; **PDBTitle:** the solution structure of horseshoe crab antimicrobial peptide2 tachystatin b with the inhibitory cystine-knot motif **PDB Entry:** PDBe RCSB PDBj) |
| 77 | c1eyoA\_ | --- \| --- \| --- | not modelled | 10.7 | 67 |


 (**PDB header:**toxin **Chain:** A: **PDB Molecule:**conotoxin tviia; **PDBTitle:** solution structure of conotoxin tviia from conus tulipa **PDB Entry:** PDBe RCSB PDBj) |
| 78 | c1v91A\_ | --- \| --- \| --- | not modelled | 13.4 | 75 |


 (**PDB header:**toxin **Chain:** A: **PDB Molecule:**delta-palutoxin it2; **PDBTitle:** solution structure of insectidal toxin delta-paluit2-nh2 **PDB Entry:** PDBe RCSB PDBj) |
| 79 | c6xyiA\_ | --- \| --- \| --- | not modelled | 10.0 | 60 |


 (**PDB header:**toxin **Chain:** A: **PDB Molecule:**ams9.3.1; **PDBTitle:** nmr solution structure of alpha-anmtx- ms11a-3 (ms11a-3) **PDB Entry:** PDBe RCSB PDBj) |
| 80 | c6xyhA\_ | --- \| --- \| --- | not modelled | 9.9 | 60 |


 (**PDB header:**toxin **Chain:** A: **PDB Molecule:**ams3; **PDBTitle:** nmr solution structure of alpha-anmtx-ms11a-2 (ms11a-2) **PDB Entry:** PDBe RCSB PDBj) |
| 81 | d1e4ra\_ | --- \| --- \| --- | not modelled | 9.6 | 50 | **Fold:** Defensin-like **Superfamily:** Defensin-like **Family:** Defensin **PDB entry:** PDBe RCSB PDBj |
| 82 | c7lyuA\_ | --- \| --- \| --- | not modelled | 15.9 | 35 |


 (**PDB header:**signaling protein **Chain:** A: **PDB Molecule:**reelin; **PDBTitle:** reelin repeat 8 **PDB Entry:** PDBe RCSB PDBj) |
| 83 | c2kraA\_ | --- \| --- \| --- | not modelled | 11.9 | 35 |


 (**PDB header:**contractile protein **Chain:** A: **PDB Molecule:**prokineticin bv8; **PDBTitle:** solution structure of bv8 **PDB Entry:** PDBe RCSB PDBj) |
| 84 | c1imtA\_ | --- \| --- \| --- | not modelled | 10.9 | 35 |


 (**PDB header:**toxin **Chain:** A: **PDB Molecule:**intestinal toxin 1; **PDBTitle:** mamba intestinal toxin 1, nmr, 39 structures **PDB Entry:** PDBe RCSB PDBj) |
| 85 | d1ayja\_ | --- \| --- \| --- | not modelled | 12.8 | 56 | **Fold:** Knottins (small inhibitors, toxins, lectins) **Superfamily:** Scorpion toxin-like **Family:** Plant defensins **PDB entry:** PDBe RCSB PDBj |
| 86 | c1fu3A\_ | --- \| --- \| --- | not modelled | 9.7 | 63 |


 (**PDB header:**metal transport inhibitor **Chain:** A: **PDB Molecule:**delta-conotoxin txvia; **PDBTitle:** three-dimensional structure in solution of the sodium channel2 agonist/antagonist delta-conotoxin txvia **PDB Entry:** PDBe RCSB PDBj) |
| 87 | d1fu3a\_ | --- \| --- \| --- | not modelled | 9.7 | 63 | **Fold:** Knottins (small inhibitors, toxins, lectins) **Superfamily:** omega toxin-like **Family:** Conotoxin **PDB entry:** PDBe RCSB PDBj |
| 88 | c5znuA\_ | --- \| --- \| --- | not modelled | 16.8 | 83 |


 (**PDB header:**toxin **Chain:** A: **PDB Molecule:**omega-conotoxin-like bu8; **PDBTitle:** structure of omega conotoxin bu8 **PDB Entry:** PDBe RCSB PDBj) |
| 89 | c1mvjA\_ | --- \| --- \| --- | not modelled | 16.2 | 83 |


 (**PDB header:**neurotoxin **Chain:** A: **PDB Molecule:**svib; **PDBTitle:** n-type calcium channel blocker, omega-conotoxin mviia nmr, 152 structures **PDB Entry:** PDBe RCSB PDBj) |
| 90 | c1cnnA\_ | --- \| --- \| --- | not modelled | 16.0 | 83 |


 (**PDB header:**toxin **Chain:** A: **PDB Molecule:**omega-conotoxin mviic; **PDBTitle:** omega-conotoxin mviic from conus magus **PDB Entry:** PDBe RCSB PDBj) |
| 91 | c1omnA\_ | --- \| --- \| --- | not modelled | 15.7 | 83 |


 (**PDB header:**neurotoxin **Chain:** A: **PDB Molecule:**omega-conotoxin m vii c (m seven c); **PDBTitle:** solution structure of omega-conotoxin mviic, a high affinity of p-type2 calcium channels, using 1h nmr spectroscopy and complete relaxation3 matrix analysis **PDB Entry:** PDBe RCSB PDBj) |
| 92 | c3q8jA\_ | --- \| --- \| --- | not modelled | 14.5 | 83 |


 (**PDB header:**toxin **Chain:** A: **PDB Molecule:**asteropsin a; **PDBTitle:** crystal structure of asteropsin a from marine sponge asteropus sp. **PDB Entry:** PDBe RCSB PDBj) |
| 93 | c1v4qA\_ | --- \| --- \| --- | not modelled | 13.4 | 83 |


 (**PDB header:**toxin **Chain:** A: **PDB Molecule:**omega-conotoxin mviic; **PDBTitle:** three-dimensional solution structure of the analogue peptide of omega-2 conotoxin mviic **PDB Entry:** PDBe RCSB PDBj) |
| 94 | c2n7fA\_ | --- \| --- \| --- | not modelled | 20.6 | 71 |


 (**PDB header:**toxin **Chain:** A: **PDB Molecule:**muo-conotoxin mfvia; **PDBTitle:** nmr solution structure of muo-conotoxin mfvia **PDB Entry:** PDBe RCSB PDBj) |
| 95 | c1rmkA\_ | --- \| --- \| --- | not modelled | 19.7 | 71 |


 (**PDB header:**toxin **Chain:** A: **PDB Molecule:**mu-o-conotoxin mrvib; **PDBTitle:** solution structure of conotoxin mrvib **PDB Entry:** PDBe RCSB PDBj) |
| 96 | d1rmka\_ | --- \| --- \| --- | not modelled | 19.7 | 71 | **Fold:** Knottins (small inhibitors, toxins, lectins) **Superfamily:** omega toxin-like **Family:** Conotoxin **PDB entry:** PDBe RCSB PDBj |
| 97 | d3c8ya1 | --- \| --- \| --- | not modelled | 16.1 | 71 | **Fold:** Fe-only hydrogenase **Superfamily:** Fe-only hydrogenase **Family:** Fe-only hydrogenase **PDB entry:** PDBe RCSB PDBj |
| 98 | c3lx4B\_ | --- \| --- \| --- | not modelled | 19.3 | 57 |


 (**PDB header:**oxidoreductase **Chain:** B: **PDB Molecule:**fe-hydrogenase; **PDBTitle:** stepwise [fefe]-hydrogenase h-cluster assembly revealed in the2 structure of hyda(deltaefg) **PDB Entry:** PDBe RCSB PDBj) |
| 99 | d1hfel1 | --- \| --- \| --- | not modelled | 11.0 | 57 | **Fold:** Fe-only hydrogenase **Superfamily:** Fe-only hydrogenase **Family:** Fe-only hydrogenase **PDB entry:** PDBe RCSB PDBj |


|  |  |
| --- | --- |
| Binding site prediction |  |

The confidence in the top model (c6av8A\_ 88.4% confidence) was considered too low (<90%) for submission to 3DLigandSite

You may manually submit your model to 3DLigandSite, but please be aware that results may be misleading

---

Phyre is now FREE for commercial users!

All images and data generated by Phyre2 are free to use in any
publication with acknowledgement

**Please cite:** The Phyre2 web portal for protein modeling, prediction and analysis.

Kelley LA *et al.*. *Nature Protocols*
10, 845-858 (2015)[pdf]
[Citation
link]

If you use the binding site
predictions from 3DLigandSite, **please also cite:**

3DLigandSite: predicting ligand-binding sites using similar structures.

Wass MN, Kelley LA and Sternberg
MJ *Nucleic Acids Research* 38, W469-73 (2010) [PubMed]

© Structural Bioinformatics Group

Imperial College London

Lawrence
Kelley, Michael Sternberg

Disclaimer

Terms and Conditions

|  |
| --- |
| **Component software** |
| Template detection: HHpred 1.51 |
| Secondary structure prediction: Psi-pred 2.5 |
| Disorder prediction: Disopred 2.4 |
| Transmembrane prediction: Memsat\_SVM |
| Multi-template modelling and *ab initio*: Poing 1.0 |
